# Characterization of Brain Lysosomal Activities in *GBA*-Related and Sporadic Parkinson’s Disease and Dementia with Lewy Bodies

**DOI:** 10.1007/s12035-018-1090-0

**Published:** 2018-06-08

**Authors:** Tim E. Moors, Silvia Paciotti, Angela Ingrassia, Marialuisa Quadri, Guido Breedveld, Anna Tasegian, Davide Chiasserini, Paolo Eusebi, Gonzalo Duran-Pacheco, Thomas Kremer, Paolo Calabresi, Vincenzo Bonifati, Lucilla Parnetti, Tommaso Beccari, Wilma D. J. van de Berg

**Affiliations:** 1 Amsterdam Neuroscience, department of Anatomy and Neurosciences, section Clinical Neuroanatomy and Biobanking, VU University Medical Center, O2 building, room 13 W01, De Boelelaan 1108, 1081 HZ Amsterdam, The Netherlands; 2 Department of Pharmaceutical Sciences, University of Perugia, Perugia, Italy; 3 Department of Clinical Genetics, Erasmus Medical Center, Rotterdam, The Netherlands; 4 Department of Medicine- section Neurology, University of Perugia, Perugia, Italy; 5 Roche Innovation Center- F. Hoffmann-La Roche Ltd, Roche Pharmaceutical Research and Early Development, Basel, Switzerland

**Keywords:** GBA variants, Autophagy-lysosomal pathway, Cathepsin D, β-hexosaminidase

## Abstract

Mutations in the *GBA* gene, encoding the lysosomal hydrolase glucocerebrosidase (GCase), are the most common known genetic risk factor for Parkinson’s disease (PD) and dementia with Lewy bodies (DLB). The present study aims to gain more insight into changes in lysosomal activity in different brain regions of sporadic PD and DLB patients, screened for *GBA* variants. Enzymatic activities of GCase, β-hexosaminidase, and cathepsin D were measured in the frontal cortex, putamen, and substantia nigra (SN) of a cohort of patients with advanced PD and DLB as well as age-matched non-demented controls (*n* = 15/group) using fluorometric assays. Decreased activity of GCase (− 21%) and of cathepsin D (− 15%) was found in the SN and frontal cortex of patients with PD and DLB compared to controls, respectively. Population stratification was applied based on *GBA* genotype, showing substantially lower GCase activity (~ − 40%) in *GBA* variant carriers in all regions. GCase activity was further significantly decreased in the SN of PD and DLB patients without *GBA* variants in comparison to controls without *GBA* variants. Our results show decreased GCase activity in brains of PD and DLB patients with and without *GBA* variants, most pronounced in the SN. The results of our study confirm findings from previous studies, suggesting a role for GCase in *GBA*-associated as well as sporadic PD and DLB.

**Electronic supplementary material:**

The online version of this article (10.1007/s12035-018-1090-0) contains supplementary material, which is available to authorized users.

## Introduction

Converging evidence from genetic, pathological, and experimental studies has suggested that impairment of the autophagy-lysosomal pathway (ALP) is a key pathological event in the pathogenesis of Parkinson’s disease (PD) and dementia with Lewy bodies (DLB) [1, 2]. Mutations in numerous genes encoding for ALP components have been associated with increased risk to develop PD, including Glucosidase Beta Acid 1 (*GBA*) [3]. Homozygous *GBA* mutations cause Gaucher disease (GD), and heterozygous *GBA* mutations form the major genetic risk factor for PD and DLB. The prevalence of *GBA* variants is estimated 5–10% in PD and DLB patients and higher in certain studied populations, particularly among Ashkenazi Jews (~ 20%) [4–6]. In GD, approximately 300 different *GBA* mutations have been described, many of which are also found in PD [7]. Recent studies showed *GBA* mutations impact the clinical phenotype of PD and DLB, as their presence has been associated with rapid eye movement sleep behavior disorder, a more rapid disease course, earlier age of onset, and higher risk for cognitive decline and dementia [8–11]. Moreover, a worse outcome has been reported for pathogenic mutations, for instance L444P, than for milder *GBA* variants such as N370S [11–14].

*GBA* encodes for the lysosomal hydrolase β-glucocerebrosidase (GCase), which catalyzes the conversion of glucosylceramide into glucose and ceramide [5]. Decreased protein levels and enzymatic activity of GCase were found in affected brain regions [15–18], dried blood spots [19], fibroblasts [20], peripheral blood mononuclear cells [21], and CSF [22–24] of PD patients with *GBA* mutations, but also in sporadic PD patients. Alterations in the activities of several other lysosomal enzymes were found in brain tissue and CSF of PD or DLB patients compared to controls, including β-hexosaminidase (β-Hex) and cathepsin D (CathD) [22–26]. In addition, decreased protein levels have been demonstrated for various other ALP components in the PD brain [27–30]. Together, these findings suggested that GCase dysfunction as well as more widespread deregulation of the lysosomal system is involved in PD and DLB pathology.

This study aims to obtain more insight into the changes in lysosomal enzymes, including GCase, in different regions of PD and DLB brains and their relation to the presence of different *GBA* variants. In order to do this, we measured enzymatic activities for GCase, CathD, and β-Hex in the frontal cortex (FC), putamen, and substantia nigra (SN) of a clinically and pathologically well-characterized cohort of PD and DLB patients, as well as in age-matched non-demented controls. To investigate whether fluctuations in enzymatic activities were reflected at the level of mRNA, we measured expression levels for *GBA*, and genes encoding CathD (*CTSD*), GCase’s protein interactors lysosomal integral membrane-protein 2 (LIMP-2) and saposin C (SapC) —which are important determinants for GCase activity [31]—as well as selected other components of the ALP. Our results confirm the involvement of GCase in *GBA*-associated as well as idiopathic PD and DLB, most pronounced in the SN, and demonstrate a stepwise decrease in GCase activity when comparing distinctive *GBA* genetic subgroups.

## Methods

### Selection of Postmortem Human Brain Tissue

Human postmortem brain tissue was collected from clinically diagnosed and neuropathologically verified advanced PD and DLB patients (15 per group) and 15 age-matched non-neurological control subjects from the Netherlands Brain Bank (NBB, Amsterdam, The Netherlands; Table 1). All donors had short postmortem delay (PMD; < 10 h), a pre-analytical factor that can potentially impact the stability of lysosomal enzymes [32]. In compliance with all local ethical and legal guidelines, informed consent for brain autopsy and the use of brain tissue and clinical information for scientific research was given by either the donor or the next of kin. Brains were dissected in compliance with standard operating protocols of the Netherlands Brain Bank (www.brainbank.nl). Frozen tissue blocks of the superior frontal cortex, putamen and SN were collected at autopsy. Tissue blocks were pulverized using a mixer mill (model MM400; Retsch, Haan, Germany) during 2 min at a frequency of 30 Hz. Pulverization took place in stainless-steel grinding jars, precooled in liquid nitrogen to prevent thawing of the tissue. The procedure was repeated, when needed, until all tissue was pulverized. Subsequently, tissue was stored at − 80 °C in aliquots.

**Table 1:** Cohort demographics. *p < 0.05 (Pearson chi-square test)

	Controls (*N* = 15)	PD (*N* = 15)	DLB (*N* = 15)	*p* value
Age of death (years ± SD)	77 ± 6	77 ± 5	77 ± 5	0.67
Sex (M/F)	4/11	11/4	10/5	0.02*
Postmortem delay (hours ± SD)	6.3 ± 1.7	6.0 ± 1.8	5.2 ± 1.2	0.47
Braak Lewy Body Score	0–1 (14/1)	4–6 (3/5/7)	4–6 (3/3/9)	< 0.01*
Braak score for Neurofibrillary Tangles	0–2 (2/6/7)	0–3 (2/9/2/2)	0–3 (1/8/5/1)	0.46
CERAD Amyloid Plaque Score	0-B (4/7/4)	O-C (7/5/2/1)	O-C (3/3/8/1)	0.21
Disease duration (years ± SD)	–	15 ± 7	6 ± 3	< 0.01*
No. of donors with *GBA* risk factors	2	3	4	–
No. of donors with pathogenic *GBA* variants	0	3	0	–

### Genotyping

High molecular-weight genomic DNA was extracted from frozen cerebellum specimens using the Gentra Puregene Tissue Kit (Qiagene, Hilden, Germany). The genomic region encompassing *GBA* open reading frame was amplified in large fragments in order to avoid amplification of the neighboring *GBAP* pseudogene. Subsequently, we Sanger sequenced *GBA* in 43 samples, as for 2 samples (1 control, 1 DLB patient), no brain tissue was available. We report variants if they are of coding effect or within 20 bp from the intron-exon boundaries, and if their minor allele frequency (MAF) is lower than 1% in Exome Aggregatium Consortium browser (ExAC, http://exac.broadinstitute.org/). Variants nomenclature is assigned according to NM_000157.3 transcript, consisting of 11 exons and encoding for a total of 536 amino acids. We also report common nomenclature attributed to *GBA* variants (between brackets), in which the amino acid count does not include the initial 39 amino acids corresponding to the residue signal peptide. We investigated the effect on splicing of *GBA* c.762-18T>A variant according to five splicing prediction tools (SpliceSiteFinder-like, MaxEntScan, NNSPLICE, GeneSplicer, and Human Splicing Finder) integrated in Alamut Visual version 4.2 (Interactive Biosoftware, Rouen, France).

### Enzyme Activity Assays

Pulverized tissue (approximately 50 mg) was lysed in a 50 mM sodium/phosphate (Na/P), 150 mM NaCl buffer, pH 7.0 and homogenized using a homogenizer (Ultra-turrax, model T10B; IKA, Wilmington, NC, USA), after which 0.1% of tergitol-typenonyl phenoxypolyethoxylethanol 40 (Igepal CA630; Sigma-Aldrich, Saint Louis, MO, USA) was added. Samples were ultra-sonicated (Sonopuls, model: UW3100; Bandelin, Berlin, Germany), kept on ice for 30 min, and subsequently centrifuged at a speed of 15,000×*g* for 10 min in a bench centrifuge (model 5415D; Eppendorf, Hamburg, Germany). Fluorometric enzyme activity assays (EAAs) for GCase, β-Hex, and CathD were performed in accordance with the protocols previously described [16, 25]. All EAAs were performed in triplicate. Mean intra-assay coefficients of variation (CV) were lower than 5% for all enzymes, with a maximum CV of 8.5%. Enzymatic activities were normalized for total protein content of the samples to obtain the specific activity. Total protein concentration in the samples was determined according to the method described by Bradford [33].

### RNA Extraction and Quantitative PCR

To determine whether fluctuations in enzymatic activities were reflected at the level of mRNA, we have measured the expression levels for different ALP-related genes using quantitative PCR (qPCR). Experiments were performed on pulverized FC and SN tissue, but not on putamen as not enough material was left. Apart from *GBA*, mRNA expression levels were measured for the genes that encode CathD and GCase’s protein interactors LIMP-2 (*SCARB2*) and saposin C (*PSAP*) [31]. Finally, genes encoding lysosomal membrane-associated receptors (*LAMP-1*; *LAMP-2*) and autophagy (*TFEB*) were measured as more general markers for the ALP. RNA extraction was performed as previously described, using a Trizol/chloroform protocol [34]. The RNA integrity number (RIN) was determined using an Agilent TM 2100 Bioanalyzer and an RNA 6000 Nano LabChip Kit (Agilent Technologies, Palo Alto, CA, USA). We applied an RIN value of 5.0 as threshold for inclusion for the qPCR analysis. Consequently, a smaller subset of 29 samples was included for the FC (11 controls/10 PD/ 8 DLB) and 27 for the SN (10 controls/9 PD/8 DLB). cDNA synthesis was done using the High-Capacity cDNA Reverse Transcription Kit (Art. No. 4368814; ThermoFischer, Waltham, MA, USA), and cDNA was stored at − 20 °C until use. For all genes of interest, intron-spanning Taqman assays were designed (Table S1). Primers were synthesized by Eurogentec (Luik, Belgium). Taqman probes were selected from the Human Universal Probe Library (UPL; Roche Applied Science, Indianapolis, IN, USA). For normalization, the housekeeping genes *m-RIP*, *OAZ-1*, and *POL2RF* were selected using geNorm software from a subset of candidate housekeeping genes [35]. Standard curves were generated for each assay run. The relative expression ratio of each target gene was calculated using the efficiency-corrected delta-delta Cq method [34, 36]. All assays were performed in duplicate (mean CV = 5.4%).

### Statistics

Hierarchical clustering analysis was done using R software (Version 3.2.5, R Foundation for Statistical Computing, Vienna, Austria) [37], to compare lysosomal enzyme activities across different brain areas. Further statistical analyses were performed using SPSS package version 20.0 (Statistical Product and Service Solutions). As enzyme activities between brain regions were correlated within subjects, general linear models for mixed effects were used to test for differences between PD and DLB patients with controls, in which contribution of individual patient groups per brain region were further studied. In other comparisons, ANCOVA analyses were applied. In all models, age was included as a covariate. For the qPCR data, the RIN value was included as weighted least squares weight to the models. A *p* value < 0.05 was considered significant in all analyses. All reported *p* values were corrected for multiple comparisons: multiple comparisons were taken into account in the general linear model for mixed effects, while Bonferroni post hoc tests were applied in the univariate models. Graphs and figures were generated using R software and GraphPad (Version 7.0, Prism, La Jolla, CA, USA) and composed in Adobe Photoshop (version CS6, Adobe Systems Incorporated, San Jose, CA).

## Results

### Lysosomal Enzyme Activities

**Fig. 1:**
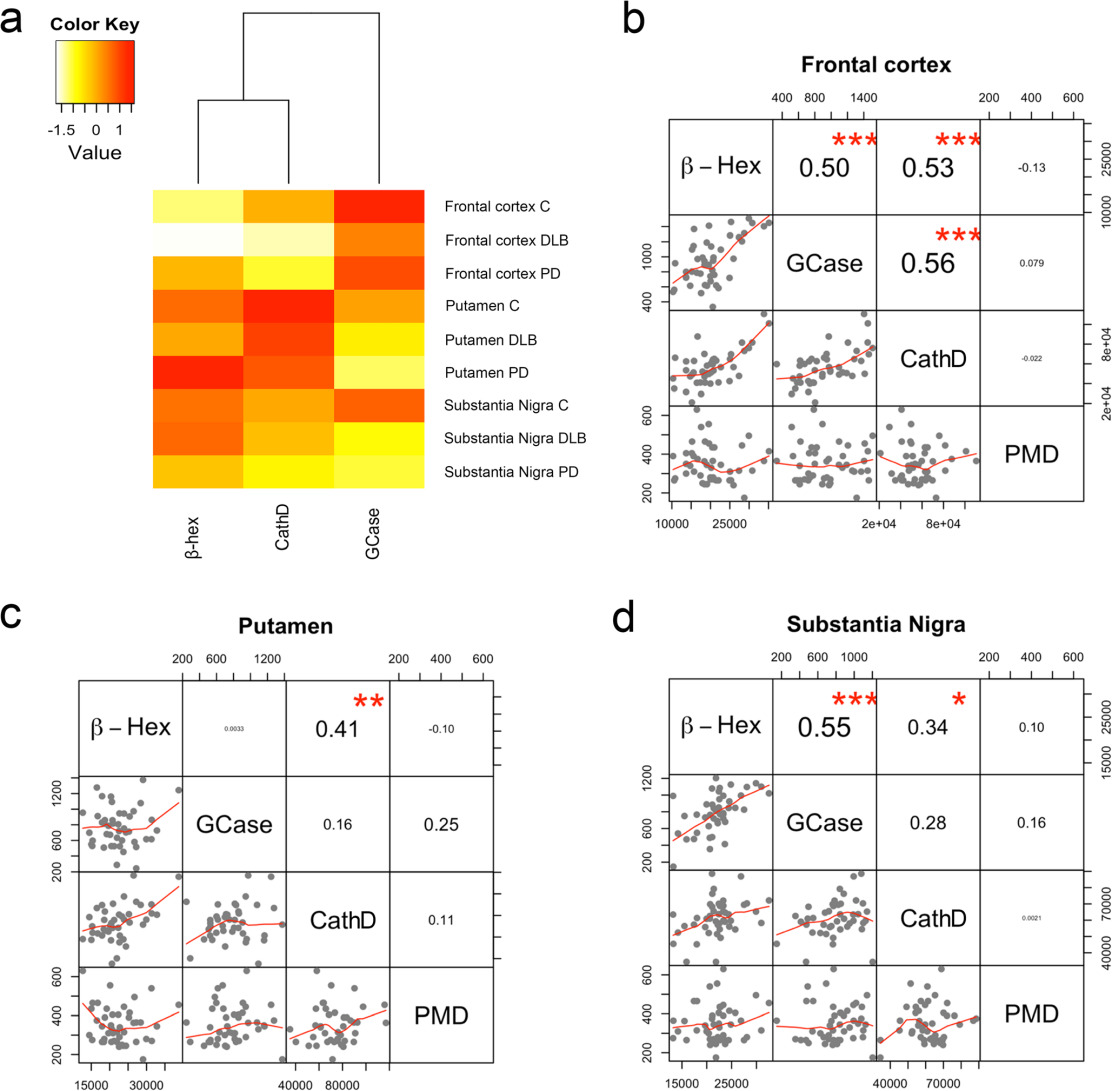
Lysosomal enzymatic activities in different regions of the human postmortem brain. **a** Cluster analysis of average enzymatic activities across patients for GCase, β-Hex, and CathD in the different studied regions reveals that GCase activity was highest in the frontal cortex, while CathD and β-Hex activities were higher in the putamen compared to the other studied regions. **b**, **c**, and **d** Correlation matrices for lysosomal enzymatic activities and PMD for frontal cortex (**b**), putamen (**c**), and SN (**d**). High correlations were sometimes observed between activities of different lysosomal enzymes, but not with PMD. Values represent Spearman’s correlation coefficients. *p < 0.05; **p < 0.01; ***p < 0.001

**Fig. 2:**
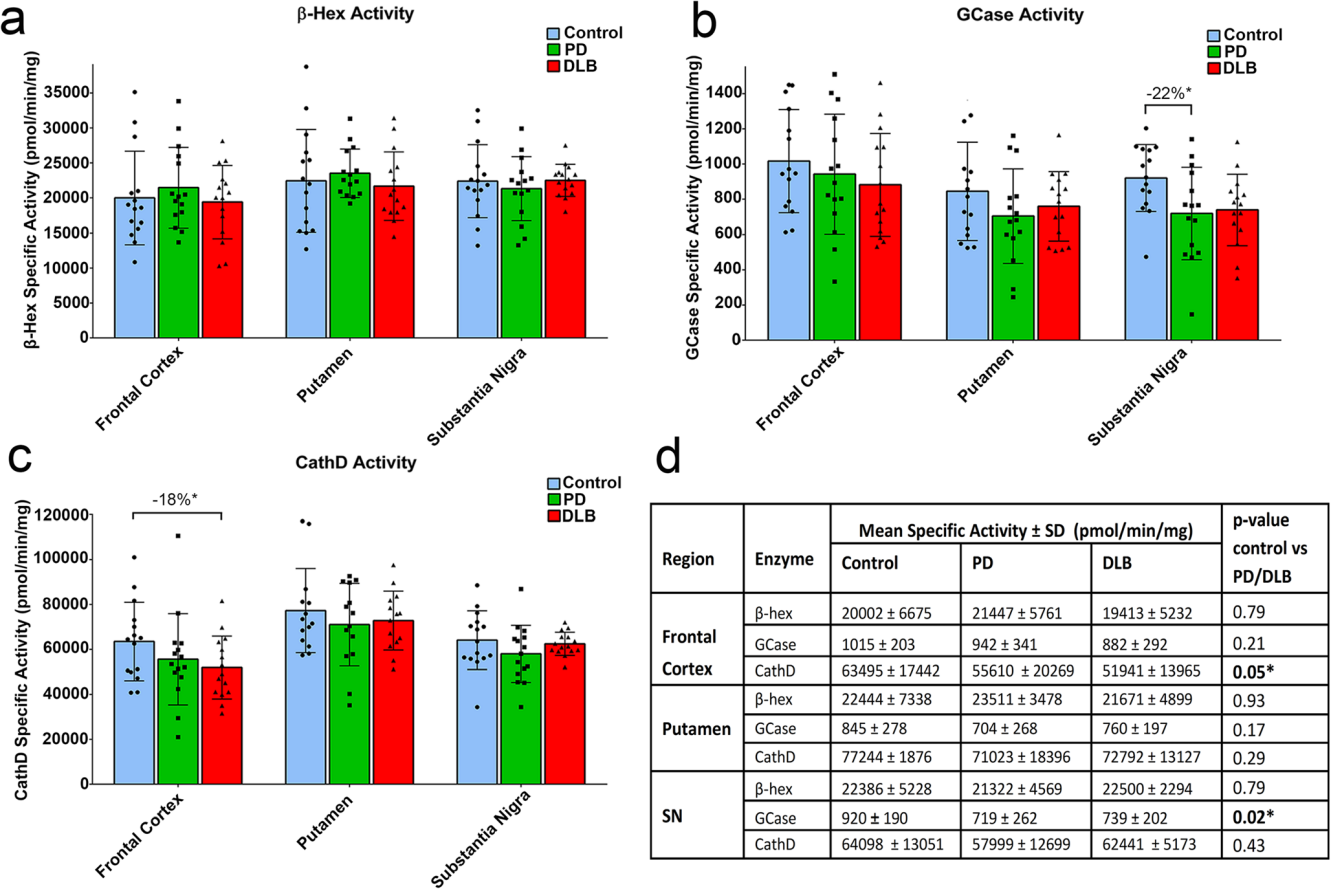
Lysosomal enzyme activities in the brain of PD and DLB patients compared to controls. Group comparison of measured specific activities for β-Hex (**a**), GCase (**b**), and CathD (**c**) expressed in pmol/min/mg of proteins. Graphs show the average specific activity and standard deviation for non-demented controls (blue), PD patients (green), and DLB patients (blue). **d.** Overview of mean enzyme activities ± standard deviations. The *p* value indicates the between-group difference per brain region. GCase activity was significantly decreased in the SN of PD patients compared to controls, while lower CathD activity was found in the frontal cortex of DLB patients compared to controls *p < 0.05

Lysosomal enzyme activities across samples revealed differences between brain regions (Fig. 1a). GCase activity was higher in the FC compared to the other regions, while CathD and β-Hex activities were highest in the putamen. Activities of different lysosomal enzymes were sometimes correlated within a brain region, but not with postmortem delay (Fig. 1b–d). GCase activity was significantly decreased in the SN of PD and DLB patients compared to controls (− 21%; *p* = 0.02), while this effect was not significant in other studied brain regions (Fig. 2b, d). Post hoc analyses revealed that GCase was particularly decreased in the SN of PD patients compared to controls (− 22%; *p* = 0.04), with a similar trend in DLB patients (− 20%; *p* = 0.06). No differences in GCase activity were observed between PD and DLB patient groups in any of the studied brain regions. For CathD, a significant decrease in enzymatic activity was found in the FC of PD and DLB patients compared to controls (− 15%; *p* = 0.05; Fig. 2c). This effect was most pronounced in DLB patients (− 18%; *p* = 0.04) while a similar trend did not reach significance in PD patients (− 12%; *p* = 0.16). For the activity of β-Hex, no between-group differences were observed in any of the measured brain regions (Fig. 2a).

### Gene Expression

**Fig. 3:**
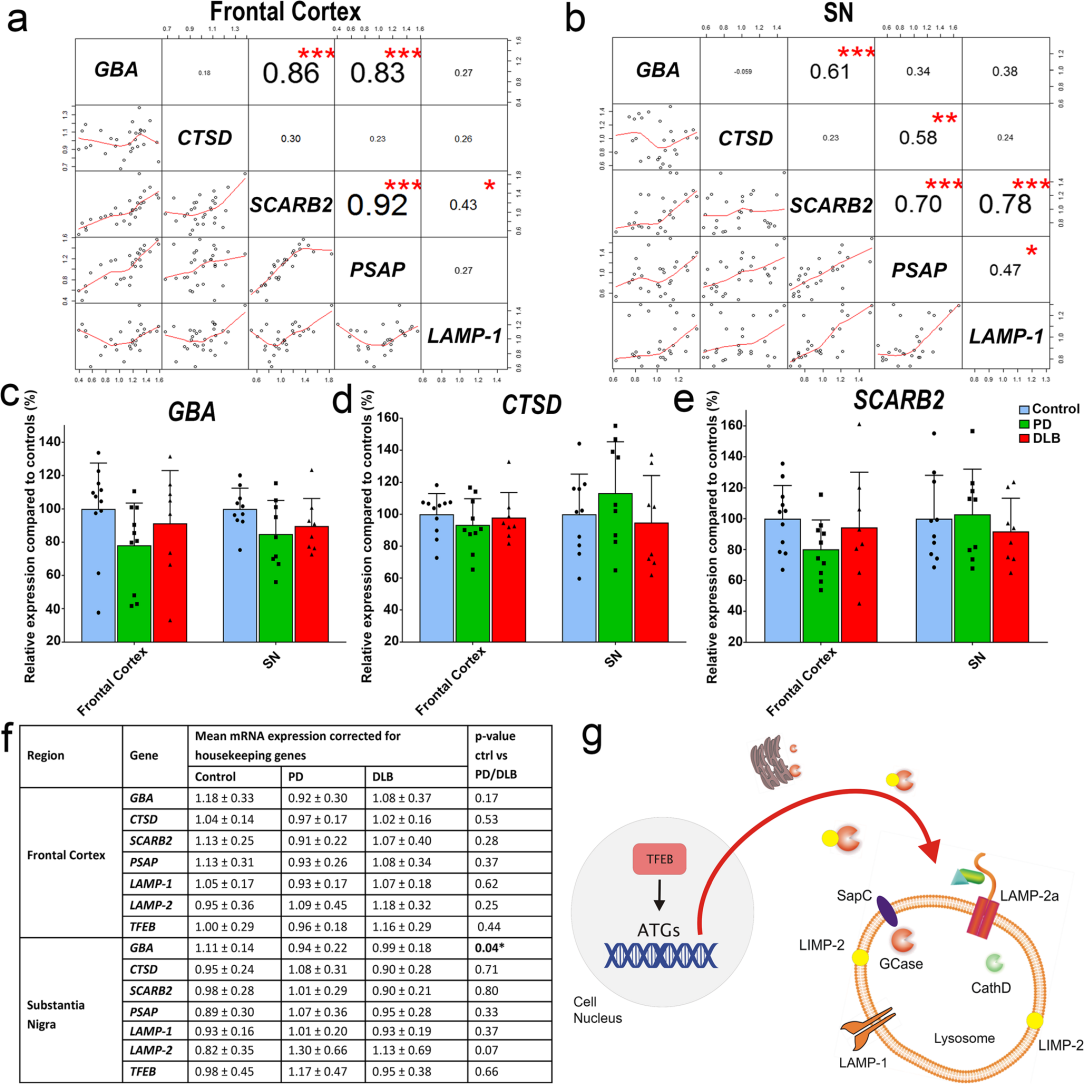
mRNA expression levels for *GBA*, *CTSD*, and other ALP components. Correlation matrices showing association between mRNA expression levels of *GBA* and genes encoding *CTSD*, GCase’s protein interactors LIMP-2 (*SCARB2*) and saposin C (*PSAP*), and the lysosomal membrane-associated protein *LAMP-1* in the frontal cortex (**a**) and SN (**b**). Normalized mRNA expression levels for *GBA* (**c**), *CTSD* (**d**), and *SCARB2* (**e**) in PD (green) and DLB (red) patients as well as non-demented controls (blue). Expression levels are expressed as percentage of the average expression level of non-demented controls. **f** Schematic outline of the interplay between the measured gene products. LIMP-2 functions as a transporter molecule for GCase from the endoplasmatic reticulum, while the interaction of GCase with SapC is essential for its proper function [31]. LAMP-1 and LAMP-2a are lysosome-associated membrane proteins, while transcription factor EB (TFEB) is the master regulator of biogenesis and function of lysosomes, by activating transcription of autophagy-related genes (ATGs). *p < 0.05; **p < 0.01; ***p < 0.001

To examine whether changes in enzyme activity were reflected at the mRNA level, gene expression was assessed for *GBA* and *CTSD*. In addition, mRNA expression levels were measured for genes encoding the GCase protein interactors LIMP-2 (*SCARB2*) and saposin C (*PSAP*), which are important determinants for GCase activity [31]. Finally, gene expression levels were measured for more general markers associated with lysosomes (*LAMP-1*; *LAMP-2*) or the ALP (*TFEB*; Fig. 3g). *GBA* and *CTSD* expression were not correlated with GCase or CathD activity in both SN and FC (Fig. S1). However, strong correlations between mRNA expression levels were found between certain ALP genes, for instance between *GBA* and *SCARB2* (Fig. 3a). Normalized mRNA expression levels for all measured genes showed substantial variability within groups. Still, decreased normalized *GBA* mRNA expression levels were observed in the SN of PD and DLB patients compared to controls (− 13%; *p* = 0.04; Fig. 3a, g). A similar pattern could be observed in the FC, although differences were not significant (− 16%; *p* = 0.17). No significant differences in the mRNA expression levels were found for *CTSD* or any of the other measured ALP-related genes in either FC or the SN (Fig. 3c, f).

### GBA Screening

According to above-mentioned criteria, six *GBA* variants were detected in a total of 12 out of 43 tested specimens, and specifically in 6 PD patients, 4 DLB patients and 2 non-demented controls (Table 1). Based on previous literature as well as on the predicted effect on GCase functioning, a distinction was made between putative pathogenic variants—that are causative for GD—and variants that are risk factors for PD. We identified *GBA* c.535G>C p.Asp179His (p.Asp140His) and *GBA* c.1448T>C p.Leu483Pro (p.Leu444Pro) variants in heterozygous state in ID-24 and ID-26 PD donors, respectively. In the context of recessive inheritance, both variants have been associated with GD and with PD if carried in heterozygous state [38]. In ID-18 (PD), we identified a *GBA* c.1073C>T p.Pro358Leu (p.Pro319Leu) variant, which has been reported before in a Dutch patient with GD type 1 [39]. As this variant affects an evolutionary-conserved amino acid, it is likely pathogenic [40].

Apart from these GD-associated pathogenic mutations, we detected variants in our cohort for which the association with GD is less clear. First, we detected heterozygous *GBA* c.1093G>A p.Glu365Lys (p.Glu326Lys) and *GBA* c.1223C>T p.Thr408Met (p.Thr369Met) variants in eight (3 PD and 3 DLB patients, 2 controls) and two (1 PD and 1 DLB patients) brain specimens, respectively. Both of these variants are not causative for GD in homozygous carriers, although they may modify GCase activity and GD phenotype [41, 42]. In particular, p.Glu326Lys has been established as risk factor for PD, as it is the most prevalent PD-associated *GBA* mutation, which was demonstrated in a large meta-analysis combining data from different genome-wide association studies [43]. Although the association of p.Thr369Met variants with PD is more controversial, accumulating evidence suggests that this variant may be risk factor with a minor effect [41]. Therefore, both p.Glu326Lys and p.Thr369Met were considered as *GBA* risk factors in further analyses [6, 10, 41]. Finally, we found *GBA* c.762-18T>A variant in homozygous state in ID-16 PD patient’s brain. Yet, this variant is not reported to be associated with GD nor PD. Out of 5 addressed splicing prediction tools, only the Human Splicing Finder prediction software predicts that the alternative nucleotide introduces a weaker acceptor site, but without affecting the efficiency of the default one. Based on these observations, we have considered the c.762-18T>A as potential PD risk factor rather than pathogenic variant in our analysis. Of note, for all these detected *GBA* PD risk factors, although MAFs are lower than 1% in the ExAc database (p.Glu326Lys, 0.98%; p.Thr369Met, 0.66%; c.762-18T>A, 0.79%), their occurrence in the Netherlands is higher than 1%, as shown by the GoNL database (p.Glu326Lys, 2.3%; p.Thr369Met, 1.1%; c.762-18T>A, 1.2%) [44].

One PD donor (ID-24) was found to carry three *GBA* alterations, namely, c.535G>C p.Asp179His (p.Asp140His), c.1093G>A p.Glu365Lys (p.Glu326Lys), and c.1223C>T p.Thr408Met (p.Thr369Met) variants. Unfortunately, the phase of these variants (cis or trans) could not be established as relatives of the carrier were not available for testing. There are examples in the literature in which multiple *GBA* variants are located on the same chromosome as the result of complex genomic rearrangements occurring between the *GBA* gene and the *GBAP* pseudogene [7], which cannot be ruled out in this case. Moreover, the co-occurrence of p.Asp140His plus p.Glu365Lys variants on complex alleles has been reported repeatedly [12, 45, 46]. Of interest, although this patient possibly met the biochemical and genetic criteria for being diagnosed with GD [47], clinically and pathologically she was referred as affected by PD.

In our study design, it was not possible to differentiate PD/DLB patients with *GBA* variants (PD/DLB+*GBA*) from PD/DLB-*GBA* patients based on clinical symptoms, mainly because of the limited sample size. However, for all *GBA* variant carriers in our study substantial cognitive symptoms and other non-motor symptoms were reported during the disease course, including visual hallucinations. An overview of prominent clinical features of the PD and DLB patients with *GBA* variants in our study is provided in Table S3. All cases with *GBA* variants showed widespread Lewy Body pathology at autopsy, with Braak α-synuclein stages similar to non-carriers in our study.

### Population Stratification Based on GBA Variants

**Fig. 4:**
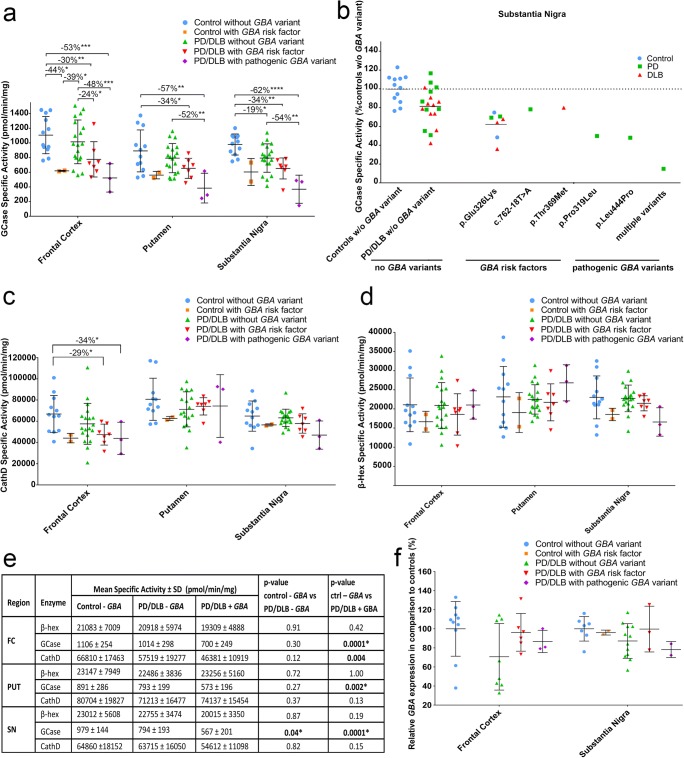
Population stratification based on *GBA* genotype. Specific enzymatic activities for GCase (**a**), β-Hex (**c**), and CathD (**d**) for non-demented controls without *GBA* variants (blue) and with *GBA* risk factors (orange), for PD and DLB patients without *GBA* variants (green), with *GBA* risk factors (red), and with pathogenic *GBA* variants (purple). **b** Measured GCase activities in the SN per *GBA* variant for controls (blue), PD patients (green), and DLB patients (red). **d** Overview of mean enzyme activities ± standard deviations in different *GBA* genetic subgroups. *p* values indicate the between-group difference per brain region. **e** GBA mRNA expression levels for controls, PD and DLB patients without *GBA* variants, with *GBA* risk factors, and pathogenic *GBA* variants. *p < 0.05; **p < 0.01; ***p < 0.001; ****p < 0.0001

GCase activity was substantially lower in PD/DLB+*GBA* patients in comparison to controls without *GBA* variants (*p* < 0.0001). This effect was present in all studied brain regions (Fig. 4a–e). Moreover, GCase enzymatic activity was significantly decreased in the group of PD/DLB+*GBA* patients compared to PD/DLB-*GBA* patients, confirming that the presence of *GBA* variants was a major determinant for GCase activity in our cohort. However, the group of PD/DLB-*GBA* patient showed significantly decreased GCase activity compared to control subjects without *GBA* variants in the SN (19%; *p* = 0.04), but to lesser extent in other brain regions (Fig. 4a, b). Similar to GCase activity, CathD activity was substantially decreased in the FC of PD/DLB+*GBA* patients compared to controls without *GBA* variants (− 31%; *p* < 0.01; Fig. 4c). However, CathD activity was not significantly altered in the other brain regions. For β-Hex, no differences in enzymatic activity were observed between PD/DLB+*GBA*, PD/DLB-*GBA* patients, and controls in any of the studied brain regions (Fig. 4d). The differences in GCase activity between PD/DLB+*GBA*, PD/DLB-*GBA* patients, and controls were not reflected by alterations on the mRNA level in either FC or SN (Fig. 4f).

## Discussion

The present study provides a comprehensive measurement of enzymatic activities as well as mRNA expression levels for GCase and other ALP components in selectively vulnerable regions of patients with advanced PD patients and DLB. We found that GCase activity is decreased in postmortem brains of PD and DLB patients with and without *GBA* variants compared to age-matched controls. Thereby, we confirm findings of previous studies in brain tissue from an independent cohort of clinically and pathologically well-characterized donors with PD and DLB with short PMD. Our study further provides insight in the relation between brain GCase activity and the presence of different *GBA* variants. In particular, population stratification based on *GBA* genotype showed a genotype-dependent decrease in GCase activity (Fig. 4). Finally, we observed decreased CathD activity in the FC of PD and DLB patients compared to controls, particularly in patients carrying *GBA* mutations.

As reported in previous studies, we find the most pronounced effects in the SN, a region associated with α-synuclein pathology and other biochemical abnormalities, including mitochondrial dysfunction, oxidative stress, and neurodegeneration [15, 16]. A contribution of dopaminergic cell-death in the SN to the reduction of GCase activity in this region cannot be excluded in our study. However, we did not observe differences in total protein content—for which we corrected enzymatic activities—or in other measured components (for instance β-Hex activity) in the SN of PD and DLB patients compared to controls. Moreover, a previous study did not detect changes in GCase activity in brain regions with massive neurodegeneration, such as the amygdala of donors with Alzheimer’s disease [15].

We found a significant 19% decrease of GCase activity in the SN of patients without *GBA* mutation compared to controls, which is in line with the slightly higher (33%) [15] or lower (12%) [16] differences that were previously reported in this brain region. However, in the first study, a possible contribution of *GBA* variants in the group of sporadic PD patients cannot be ruled out as no genetic screening was reported for this group of donors [15]. Genotyping of our cohort revealed a markedly high rate of *GBA* variants, with 6 carriers out of 15 screened PD donors, 4/14 DLB donors, and 2/14 screened controls. Two previously defined pathogenic variants (Asp140His and p.Leu444Pro) were detected, while one possibly pathogenic missense variant (p.Pro319Leu) was found. The presence of *GBA* variants resulted in a substantially lower GCase activity in all studied brain regions (~ − 40%), most pronounced in the SN, where the PD/DLB+*GBA* group showed no overlap with controls (Fig. 4b).

GCase activities were lowest in donors with pathogenic *GBA* variants. Although sample size for different genetic *GBA* subgroups—particularly, the number of donors with pathogenic *GBA* variants—was small, our data indicates a stepwise decrease in GCase activity in different *GBA* genetic subgroups. Interestingly, a similar “dose-effect” of *GBA* variants in PD has been suggested in recent clinical and genetic studies, in which differential effects of mild and severe *GBA* variants on PD clinical phenotype were reported. Severe *GBA* variants were associated with more progressive forms of PD [9, 11–13], and with higher genetic risk to develop PD [14]. Insight into the effects of different *GBA* variants on GCase activity in the brain can be important for patient selection and therapeutic efficacy for novel therapies that aim to increase GCase enzyme activity as disease-modifying therapy in PD/DLB, for instance using small molecule non-inhibitory chaperones [48].

The lowest GCase activity was detected in PD donor ID-24 who carried three *GBA* variants, whose phases (*cis* or *trans*) could not be established. Therefore, it is not clear whether these three variants contribute to the lower enzymatic activity in an independent manner, with the p.Asp140His variant acting as driving factor, or if they act in a synergic fashion. The extremely low GCase enzymatic activity prompts us to speculate that both *GBA* alleles are altered, with two variants being in *cis* and the remaining one in *trans*. In particular, a combination of p.Asp140His plus p.Glu365Lys variants at one allele has been reported in several studies [12, 45, 46]. Of interest is the fact that this patient, despite of very low GCase activities, was diagnosed with PD and not with GD during her life.

To investigate whether changes in enzymatic activity were reflected at the level of mRNA, we measured expression levels of *GBA* and *CTSD*. In addition, we measured expression levels for genes encoding GCase’s protein interactors LIMP-2 and SapC—which are proposed to be important determinants for GCase activity [31], to explore their interrelation. No correlation was found between *GBA* and *CTSD* mRNA expression with GCase or CathD activity in our data, while the systematic decrease in GCase activity in different pathological and genetic *GBA* subgroups was not observed at the mRNA level. The lack of association between mRNA levels and GCase enzymatic activity was not unexpected, as it has been reported before in studies on GD [7], and indicates that GCase activity is not simply a reflection of a *GBA* gene expression but also driven by other factors. In that aspect, it is interesting that we observed strong correlations between *GBA* and genes encoding its posttranslational regulators LIMP-2 and SapC, indicating an interrelation of these components at a transcriptional level.

Gene expression data showed large variability in patients and controls. This variability may be partially explained by differences in tissue composition. In particular, the content of cellular components in diseased tissue can be changed as a result of inflammatory and neurodegenerative processes. Despite of this variability, still, a trend for decreased *GBA* mRNA expression levels was observed in the SN of PD and DLB patients. Reduced *GBA* expression in the SN has previously been reported [16], while another study reported a trend for decreased *GBA* mRNA expression in the anterior cingulate cortex [17]. In other regions, including putamen [15] and occipital cortex [17], no evidence of reduced *GBA* mRNA expression was found. Interestingly, the results after population stratification showed that reduced *GBA* expression was not associated with the presence of *GBA* variants. Taken together, we conclude that lower GCase activities do not correspond with lower mRNA levels, while a role for altered *GBA* mRNA levels in sporadic PD and DLB cannot be excluded. However, at this point, the relevance of this latter observation is not clear.

We did not find differences in β-Hex activities between PD and DLB patients compared to controls. A role for β-Hex in PD pathology has been suggested, as patients affected by GM2 gangliosidosis, which results from reduced β-Hex activity, can manifest with parkinsonism [49–51]. Further, a role of β-Hex in PD pathogenesis is supported by animal studies showing that mice lacking β-Hex showed α-synuclein-positive neuronal inclusions at 4 months of age [52]. In biomarker studies, it was reported that CSF β-Hex enzyme activity is altered in PD patients compared to age-matched neurological controls [22, 23]. However, no difference in β-Hex activity was detected in brain tissue of PD and DLB patients compared to controls, in our and also other studies [15, 16, 24–26]. Together, the evidence for a role of β-Hex in sporadic PD is currently limited.

We observed decreased CathD activity in the FC of DLB patients compared to controls, while a similar non-significant effect was observed in PD patients. Of interest, CathD activity was particularly decreased in patients with *GBA* variants. A role for CathD has been proposed in different neurodegenerative diseases [53], including Alzheimer’s disease, Huntington’s disease, and PD [54–57]. Our results are in line with a previous study, which also showed lower CathD activity in the FC of patients with PD and DLB [28], while reduced expression levels of CathD have also been reported in the SN of PD patients [27]. CathD is a lysosomal protease involved in the posttranslational cleavage of prosaposin (encoded by *PSAP*), leading to the production of saposin C, a GCase activator [31, 58]. Thus, interestingly, although we did not find changes in the mRNA expression of *PSAP*, the activity of the enzyme responsible for its posttranslational regulation was altered.

The within-subject design of our study allowed to demonstrate the differential regulation of lysosomal enzymatic activity in different brain regions, as has been suggested previously in the literature [16]. However, in contrast to this study [16], enzyme activities were not generally higher in the SN. Rather, this effect seems to depend on the specific lysosomal enzyme, as CathD activity was significantly higher in the putamen than in SN and FC. A systematic mapping of lysosomal enzyme gene/protein expression and activities in the brain could provide more insight into the differential regulation of lysosomal enzymes between brain regions, and may allow better understanding of region-specific effects of lysosomal proteins.

In conclusion, our results show decreased GCase activity in the brain of PD and DLB patients with and without *GBA* variants. GCase activities in brain tissue were related to the presence as well as the pathogenicity of *GBA* variants. The results of our study confirm findings from previous studies, and provide important insights into the role of GCase in *GBA*-associated as well as sporadic PD and DLB.

## Electronic Supplementary Material

Figure S1 (DOCX 185 KB)

Table S1 (DOCX 25.0 KB)

Table S2 (DOCX 18.7 KB)

Table S3 (DOCX 18.9 KB)

Table S4 (XLSB 34 kb)
