# Supplementary material for: Characterization of Brain Lysosomal Activities in GBA-Related and Sporadic Parkinson’s Disease and Dementia with Lewy Bodies
Source: Mol Neurobiol. 2018 Jun 8;56(2):1344–55. doi: 10.1007/s12035-018-1090-0 (PMC6400877; doi:10.1007/s12035-018-1090-0)
Supplement: Supplementary file 1 — (DOCX 185 KB) [file 12035_2018_1090_MOESM1_ESM.docx]

**Molecular Neurobiology**

**Characterization of brain lysosomal activities in *GBA*-related and sporadic Parkinson's Disease and Dementia with Lewy Bodies**

Tim E. Moors, Silvia Paciotti, Angela Ingrassia, Marialuisa Quadri, Guido Breedveld, Anna Tasegian, Davide Chiasserini, Paolo Eusebi, Gonzalo Duran-Pacheco, Thomas Kremer, Paolo Calabresi, Vincenzo Bonifati, Lucilla Parnetti, Tommaso Beccari, Wilma D.J. van de Berg.

**Corresponding author:**

Tim E. Moors, MSc

Dept. of Anatomy & Neurosciences, Section Clinical Neuroanatomy

Amsterdam Neuroscience

VU University Medical Center Amsterdam

e-mail: t.moors@vumc.nl

**Fig S1:** Correlation charts showing the limited association between mRNA expression levels of *GBA* and genes encoding *CTSD* with enzymatic activities for GCase and CathD in the frontal cortex (**A**) and SN (**B**).

**
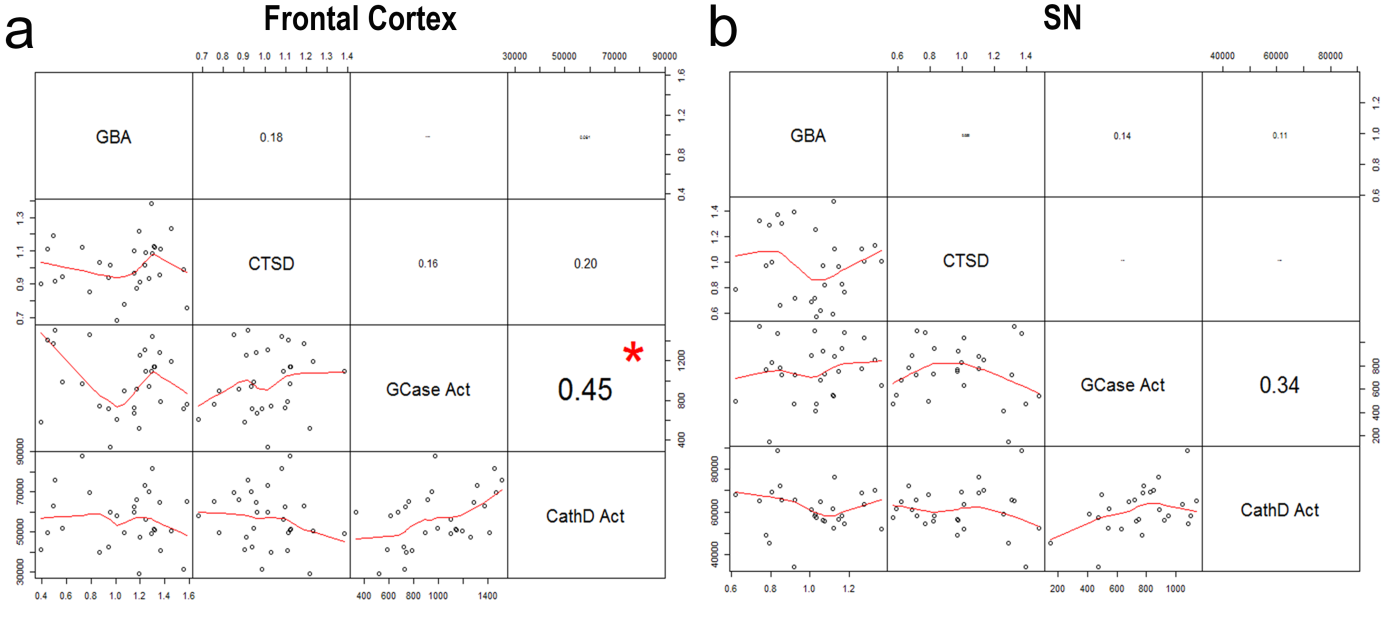
**
