# Supplementary material for: Characterization of Brain Lysosomal Activities in GBA-Related and Sporadic Parkinson’s Disease and Dementia with Lewy Bodies
Source: Mol Neurobiol. 2018 Jun 8;56(2):1344–55. doi: 10.1007/s12035-018-1090-0 (PMC6400877; doi:10.1007/s12035-018-1090-0)
Supplement: Supplementary file 2 — (DOCX 25.0 KB) [file 12035_2018_1090_MOESM2_ESM.docx]

**Molecular Neurobiology**

**Characterization of brain lysosomal activities in GBA-related and sporadic Parkinson's Disease and Dementia with Lewy Bodies**

Tim E. Moors, Silvia Paciotti, Angela Ingrassia, Marialuisa Quadri, Guido Breedveld, Anna Tasegian, Davide Chiasserini, Paolo Eusebi, Gonzalo Duran-Pacheco, Thomas Kremer, Paolo Calabresi, Vincenzo Bonifati, Lucilla Parnetti, Tommaso Beccari, Wilma D.J. van de Berg.

**Corresponding author:**

Tim E. Moors, MSc

Dept. of Anatomy & Neurosciences, Section Clinical Neuroanatomy

Amsterdam Neuroscience

VU University Medical Center Amsterdam

e-mail: t.moors@vumc.nl

**Table S1: Patient characteristics and detected GBA variants;** CD: clinical diagnosis; PMD: post-mortem delay; NFTs: neurofibrillary tangles; LB: Lewy Body; NDC: non-demented control

| **ID** | **Sex** | **Age** | **Clinical diagnosis** | **Cause of death** | **LBD Duration (yrs)** | **PMD** | **Braak**  **Score for NFTs** | **Amyloid Plaque**  **Score** | **Braak**  **LB Score** | **GBA variant** |
| --- | --- | --- | --- | --- | --- | --- | --- | --- | --- | --- |
| ***1*** | F | 77 | Control | Pulmonary metastasis | - | 02:55 | 1 | B | 0 | - |
| ***2*** | F | 84 | Control | Myelo dysplasia | - | 06:55 | 1 | O | 0 | - |
| ***3*** | F | 73 | Control | Palliative sedation | - | 07:45 | 1 | B | 0 | c.1093G>A p.Glu365Lys |
| ***4*** | F | 79 | Control | Cardiac insufficiency | - | 10:30 | 1 | O | 0 | - |
| ***5*** | F | 83 | Control | Ileus with pancreatic cancer | - | 04:40 | 1 | B | 0 | - |
| ***6*** | F | 76 | Control | Hepatic failure | - | 07:15 | 2 | O | 0 | - |
| ***7*** | M | 76 | Control | Lung cancer | - | 06:45 | 0 | O | 0 | n.a. |
| ***8*** | F | 70 | Control | Pulmonary insufficiency | - | 06:15 | 2 | A | 0 | - |
| ***9*** | F | 83 | Control | Gastroenteritis | - | 06:03 | 2 | B | 0 | - |
| ***10*** | F | 70 | Control | Cachexia | - | 07:35 | 2 | A | 0 | - |
| ***11*** | F | 64 | Control | Pneumonia | - | 05:40 | 0 | A | 0 | c.1093G>A p.Glu365Lys |
| ***12*** | F | 78 | Control | Bronchopneumonia | - | 04:35 | 2 | A | 0 | - |
| ***13*** | M | 79 | Control | Euthanasia | - | 05:45 | 2 | A | 0 | - |
| ***14*** | M | 79 | Control | Euthanasia | - | 06:30 | 2 | A | 0 | - |
| ***15*** | M | 83 | Control | Myocardial infarction | - | 05:15 | 1 | A | 1 | - |
|  |  |  |  |  |  |  |  |  |  |  |
| ***16*** | F | 80 | PD | Dehydration | 13 | 05:15 | 1 | C | 6 | c.762-18T>A |
| ***17*** | F | 81 | PD | Unknown | 5 | 06:05 | 0 | O | 5 | - |
| ***18*** | M | 84 | PD | Pneumonia, dehydration | 24 | 09:00 | 1 | A | 5 | c.1073C>T p.Pro358Leu |
| ***19*** | M | 74 | PD | Dehydration, urosepsis | 24 | 06:45 | 1 | O | 5 | - |
| ***20*** | M | 69 | PD | Respiratory insufficiency | 11 | 09:15 | 1 | A | 5 | c.1093G>A p.Glu365Lys |
| ***21*** | M | 76 | PD | Pneumonia, dehydration | 12 | 04:05 | 1 | O | 6 | c.1093G>A p.Glu365Lys |
| ***22*** | M | 81 | PD | Airway infection | 17 | 05:50 | 1 | O | 5 | - |
| ***23*** | M | 80 | PD | Cardiac failure | 13 | 05:30 | 2 | B | 6 | - |
| ***24*** | F | 83 | PD | Cachexia, dehydration | 13 | 06:05 | 1 | O | 4 | c.1093G>A p.Glu365Lys, c.535G>C p.Asp179His,  c.1223C>T p.Thr408Met |
| ***25*** | M | 72 | PD | Gastro-intestinal bleeding | 8 | 04:00 | 1 | A | 6 | - |
| ***26*** | M | 65 | PD | Dehydration | 19 | 04:25 | 1 | B | 6 | c.1448T>C p.Leu483Pro |
| ***27*** | M | 78 | PD | Pneunomia | 16 | 06:15 | 0 | A | 4 | - |
| ***28*** | M | 73 | PD | Unknown | 13 | 04:10 | 3 | O | 4 | - |
| ***29*** | F | 81 | PD | Cachexia | 33 | 05:10 | 3 | A | 6 | - |
| ***30*** | M | 76 | PD | Dehydration, cachexia | 10 | 08:15 | 2 | O | 6 | - |
|  |  |  |  |  |  |  |  |  |  |  |
| ***31*** | M | 81 | DLB | Stomach bleeding | 4 | 05:45 | 2 | B | 5 | n.a. |
| ***32*** | F | 76 | DLB | Dehydration | 10 | 04:05 | 1 | B | 6 | - |
| ***33*** | F | 72 | DLB | Dehydration | 6 | 07:25 | 1 | A | 4 | - |
| ***34*** | M | 70 | DLB | Cachexia | 6 | 04:25 | 1 | A | 6 | - |
| ***35*** | M | 83 | DLB | Dehydration / delirium | 4 | 05:10 | 2 | B | 5 | - |
| ***36*** | M | 78 | DLB | Pentobarbital overdose | 4 | 07:45 | 2 | C | 6 | - |
| ***37*** | M | 72 | DLB | Cachexia | 12 | 04:30 | 1 | O | 6 | c.1093G>A p.Glu365Lys |
| ***38*** | F | 80 | DLB | Euthanasia | 2 | 05:00 | 1 | O | 5 | c.1093G>A p.Glu365Lys |
| ***39*** | M | 75 | DLB | Dehydration | 5 | 04:25 | 2 | B | 6 | - |
| ***40*** | M | 79 | DLB | Uraemia by dehydration | 3 | 04:25 | 1 | B | 6 | - |
| ***41*** | F | 78 | DLB | Dehydration, cachexia | 9 | 04:50 | 2 | B | 6 | - |
| ***42*** | F | 81 | DLB | Cachexia | 7 | 06:30 | 1 | A | 4 | c.1223C>T p.Thr408Met |
| ***43*** | M | 69 | DLB | Pneumonia | 4 | 04:40 | 3 | B | 6 | c.1093G>A p.Glu365Lys |
| ***44*** | M | 83 | DLB | Pneumonia w. heart failure | 13 | 04:30 | 0 | O | 6 | - |
| ***45*** | M | 74 | DLB | Aspiration pneumonia | 8 | 04:25 | 1 | B | 4 | - |
