# Supplementary material for: Characterization of Brain Lysosomal Activities in GBA-Related and Sporadic Parkinson’s Disease and Dementia with Lewy Bodies
Source: Mol Neurobiol. 2018 Jun 8;56(2):1344–55. doi: 10.1007/s12035-018-1090-0 (PMC6400877; doi:10.1007/s12035-018-1090-0)
Supplement: Supplementary file 3 — (DOCX 18.7 KB) [file 12035_2018_1090_MOESM3_ESM.docx]

**Molecular Neurobiology**

**Characterization of brain lysosomal activities in *GBA*-related and sporadic Parkinson's Disease and Dementia with Lewy Bodies**

Tim E. Moors, Silvia Paciotti, Angela Ingrassia, Marialuisa Quadri, Guido Breedveld, Anna Tasegian, Davide Chiasserini, Paolo Eusebi, Gonzalo Duran-Pacheco, Thomas Kremer, Paolo Calabresi, Vincenzo Bonifati, Lucilla Parnetti, Tommaso Beccari, Wilma D.J. van de Berg.

**Corresponding author:**

Tim E. Moors, MSc

Dept. of Anatomy & Neurosciences, Section Clinical Neuroanatomy

Amsterdam Neuroscience

VU University Medical Center Amsterdam

e-mail: t.moors@vumc.nl

**Table S2: Specification of primers for qPCR.**

**Abbreviation:** UPL: Human Universal Probe Library

| **Target** | **Primer sequence**  **5’ 3’** | **UPL probe number** | **Amplicon length** | **Holding stage (95°C)** | **Denaturation (95°C)** | **Annealing (T(°C)/sec)** | **Extension (65**°C**)** |
| --- | --- | --- | --- | --- | --- | --- | --- |
| *GBA* | F: CCTACTCATGCTGGATGACCA  R: CCAATGTACAGCAATGCCATGAA | # 43 | 105 bp | 15 min | 15 sec | 58 °C/35 sec | 30 sec |
| *CTSD* | F: CTC-TGT-CCT-ACC-TGA-ATG-TCA  R: AAG-TGC-CTG-TGT-CCA-CAA-TG | # 39 | 122 bp | 15 nmin | 15 sec | 58 °C/35 sec | 40 sec |
| *LIMP-2* | F: GAAACGGGAGACATTAGAACCA  R: CAAAGTAGTGTTAATCATAGACTTCAG | # 48 | 110 bp | 15 min | 15 sec | 59 °C/35 sec | 40 sec |
| *LAMP-1* | F: GGGTCCAGGCTTTCAAGGT  R: GGCACCACCCACAGCGAT | # 25 | 100 bp | 15 min | 15 sec | 57 °C/30 sec | 35 sec |
| *LAMP-2* | F: AATGTGACACAAGGAAAGTATTCTAC  R: ATAAGCCAGCAACACTAGAAT | # 24 | 122 bp | 15 min | 15 sec | 58°C/40 sec | 45 sec |
| *TFEB* | F: GAGATGACCAACAAGCAGCTC  R: AGCTCAGCCATGTTCATGCC | # 17 | 106 bp | 15 min | 15 sec | 56 °C/30 sec | 30 sec |
| *m-RIP* | F: CTCAACGACGAGGACCTGAC  R: GGTGCACTGGGTTGTCAAA | # 36 | 93 bp | 15 min | 15 sec | 59°C/30 sec | 20 sec |
| *OAZ1* | F: CACCATGCCGCTCCTAAG  R: ACAGCAGTGGAGGGAGAC | # 74 | 75 bp | 15 min | 15 sec | 57°C/30 sec | 20 sec |
| *POL2RF* | F: CATGTCAGACAACGAGGACAA  R: TCCAAGTCATCTAGCCCTTCA | # 25 | 77 bp | 15 min | 15 sec | 60°C/35 sec | 35 sec |
