# Supplementary material for: Characterization of Brain Lysosomal Activities in GBA-Related and Sporadic Parkinson’s Disease and Dementia with Lewy Bodies
Source: Mol Neurobiol. 2018 Jun 8;56(2):1344–55. doi: 10.1007/s12035-018-1090-0 (PMC6400877; doi:10.1007/s12035-018-1090-0)
Supplement: Supplementary file 4 — (DOCX 18.9 KB) [file 12035_2018_1090_MOESM4_ESM.docx]

**Molecular Neurobiology**

**Characterization of brain lysosomal activities in *GBA*-related and sporadic Parkinson's Disease and Dementia with Lewy Bodies**

Tim E. Moors, Silvia Paciotti, Angela Ingrassia, Marialuisa Quadri, Guido Breedveld, Anna Tasegian, Davide Chiasserini, Paolo Eusebi, Gonzalo Duran-Pacheco, Thomas Kremer, Paolo Calabresi, Vincenzo Bonifati, Lucilla Parnetti, Tommaso Beccari, Wilma D.J. van de Berg.

**Corresponding author:**

Tim E. Moors, MSc

Dept. of Anatomy & Neurosciences, Section Clinical Neuroanatomy

Amsterdam Neuroscience

VU University Medical Center Amsterdam

e-mail: t.moors@vumc.nl

| **ID** | **GBA Genotype** | **CD** | **Sex** | **Age at onset (yrs)** | **Age of death (yrs)** | **L-Dopa resp.** | **Family history of PD/DLB** | **First symptom** | **(Later) motor symptoms** | **(Later) cognitive symptoms** | **Other**  **non-motor symptoms** |
| --- | --- | --- | --- | --- | --- | --- | --- | --- | --- | --- | --- |
| 16 | c.762-18T>A (VUS) | PD | F | 67 | 80 | Yes | n.a. | Walking problems; hypokinetic syndrome | Extrapyramidal symptoms;  falls; apraxia. | Variable alertness; aphasia; disorientation problems; dementia; confusion. | Visual and auditory hallucinations Behavioral problems. |
| 18 | c.1073C>T p.Pro358Leu (p.Pro319Leu; VUS) | PD | M | 60 | 84 | Yes | Negative | Mild one-sided Parkinson syndrome at the left side of the body | Freezing; walking problems; dyskinesia | Memory function decreased; word finding problems; concentration problems; confusion | Visual hallucinations; character changes |
| 20 | c.1093G>A p.Glu365Lys (p.Glu326Lys) | PD | M | 58 | 69 | Yes | n.a. | Depression; initial dementia | Bradykinesia; starting problems; shuffling gate; freezing;; falls; tremor. | Memory problems; speech; word finding problems; dementia. | Depression; frequent hallucinations; psychosis. |
| 21 | c.1093G>A p.Glu365Lys (p.Glu326Lys) | PD | M | 64 | 76 | Yes | n.a. | n.a. | Apraxia; tremor; walking problems; Falling. | Memory problems; orientation disturbed; confusion. | Incontinency; narcolepsy. Hallucinations; agitated behavior; constipation. |
| 24 | Multiple variants:  Asp140His; p.Glu326Lys; p.Thr369Met | PD | F | 70 | 83 | Yes | n.a. | Tremor; double vision; vertigo; depressive mood | Choreatic movements; stability and walking problems; falling; swallowing problems. | Memory problems; confusion; dementia. | Incontinence; constipation; visual and auditive hallucinations; sleeping problems; reduced consciousness. |
| 26 | c.1448T>C p.Leu483Pro  (p.Leu444Pro) | PD | M | 46 | 65 | No  (DBS in STN) | Yes (mother suffered from PD). | Mild one-sided Parkinson syndrome at the left side of the body. | Walking problems; falling; swallowing problems; speech problems; freezing. | Mild dementia; progressive cognitive disturbances. | Sleeping problems; excessive daytime sleepiness; behavioral problems; hallucinations. |
| 37 | c.1093G>A p.Glu365Lys (p.Glu326Lys) | DLB | M | 60 | 72 | Yes | Yes (sister; brother and aunt suffered from PD. | Memory problems. | Stooped posture; tremor; walking problems; hypokinesia; speech problems (dysarthria); falling. | Word finding and concentration problems; disturbed attention and orientation; confusion. | Constipation; behavioral problems; incontinence; hallucinations. |
| 38 | c.1093G>A p.Glu365Lys (p.Glu326Lys) | DLB | F | 78 | 80 | Yes | Negative | Memory problems | Asymmetric rigidity; soft voice | Word finding problems; impaired orientation | Constipation; pseudohallucinations |
| 42 | c.1223C>T p.Thr408Met (p.Thr369Met) | DLB | F | 74 | 81 | Yes | No (mother bipolar and demented; father demented) | Bradykinesia ; problems with speech fine motor skills and walking; Hypersalivation; Depressive mood. | Rigidity; falling. | Memory problems; concentration problems; bradyphrenia; confusion; disturbed orientation. | Depressive mood; hypersalivation; hallucinations; constipation. |
| 43 | c.1093G>A p.Glu365Lys (p.Glu326Lys) | DLB | M | 65 | 69 | n.a. | No (mother and father of mother demented) | Memory problems and apraxia | Swallowing problems. | Impaired attention; confusion; psychosis; variable consciousness; disturbed orientation in time. | Depressive mood; anxiety; sleeping problems; behavioral problems. |

**Table S3:** Clinical features of PD and DLB patients with GBA variants. Abbreviations: CD: clinical diagnosis; M/F: male/female; n.a: not available
